# Healthy Grains in Healthy Diets: The Contribution of Grain Foods to Diet Quality and Health in the National Health and Nutrition Examination Survey 2017–2023

**DOI:** 10.3390/nu17162674

**Published:** 2025-08-19

**Authors:** Adam Drewnowski, Rozenn Gazan, Matthieu Maillot

**Affiliations:** 1 Center for Public Health Nutrition, University of Washington, Seattle, WA 98195, USA; 2 MS-Nutrition, 27 bld Jean Moulin Faculté de Médecine la Timone, Laboratoire C2VN, 13385 Marseille Cedex 5, France; rozenn.gazan@ms-nutrition.com (R.G.); matthieu.maillot@ms-nutrition.com (M.M.)

**Keywords:** healthy grains, carbohydrate food quality score (CFQS-3), nutrient rich food index for grains (NRF9.3g), healthy eating index (HEI 2020), diet quality, body mass index (BMI), insulin, waist circumference, HDL cholesterol

## Abstract

**Background**: Grain foods are important sources of complex carbohydrates, fiber, vitamins, and minerals. **Objective**: To identify healthy grain foods and to assess their associations with composite diet quality measures and selected health outcomes. **Methods**: Healthy grain foods were identified using two methods. The first one, Carbohydrate Food Quality Score (CFQS-3) was based on whole grains, fiber, and added sugar. The second, NRF9.3g score for grains, balanced nutrients to encourage (protein, fiber, vitamins B1, B2, B3, and E, folate, iron, and magnesium) against added sugar, sodium, and saturated fat. Nutrient composition data for 1244 grain foods came from the USDA Food and Nutrient Database for Dietary Studies (FNDDS 2017–2023). Dietary intakes came from the National Health and Nutrition Examination Surveys (NHANES 2017–2023). The Healthy Eating Index (HEI 2020) and the diet-level Nutrient Rich Food Index (NRF) were the two measures of diet quality. National food prices came from the USDA 2021 Thrifty Food Plan. Data on body weight, waist circumference, insulin, and cholesterol came from NHANES clinical files. **Results**: Healthy grain foods were those that scored >2 points on CFQS-3 or were in the top tertile of NRF9.3g scores. The CFQS-3 score favored cooked whole grains and cereals and savory snacks. The NRF9.3g score gave the highest ratings to breads, rolls, and RTE cereals. Consumers of healthy grains identified using both methods had higher HEI 2020 values and higher diet-level NRF scores. Both effects were dose-dependent. Consumption of healthy grains was associated with lower obesity rates and lower fasting insulin levels. **Conclusions**: Consumption of healthy grain foods was associated with healthier diets and lower obesity prevalence. Dietary guidelines need to acknowledge the contribution of healthy grain foods to diet quality and health.

## 1. Introduction

Grain foods fit the definition of nutrient-rich foods, providing more nutrients than calories. Providing just 15% of total daily energy, grain foods are important sources of plant protein, fiber, folate, iron, zinc, calcium, and magnesium [1,2,3,4]. The Dietary Guidelines for Americans (DGA) 2020–2025 have recommended making half of dietary grains whole grains [1]. The Dietary Guidelines Advisory Committee (DGAC) Scientific Report for 2025–2030 went further by recommending mostly whole grains [2]. The DGAC report confirmed that diets higher in whole grains are associated with lower risk of heart disease, colorectal cancer, breast cancer, and gestational diabetes [2]. The inclusion of grain foods in dietary guidance is justified in multiple published papers [3,4,5,6].

The beneficial health effects of grain foods are not limited to whole grains. Foods made with enriched flour contain vitamins B1, B2, and B3, along with folic acid. Fortified breakfast cereals contain a variety of key vitamins and minerals, including vitamin A, B2, B6, folate, B12, vitamin D, calcium, magnesium, potassium, iron, and zinc [7,8,9]. In past analyses of NHANES data, ready-to-eat (RTE) cereals were consistently associated with improved dietary outcomes [9].

The present challenge was to assess the nutritional value of grain foods using methods that would capture nutrients, ingredients (whole grains), and fortificants (e.g., folate, iron). Quantitative methods to capture the healthfulness of foods are known under the collective name of nutrient profiling [10]. However, most methods used to assess the carbohydrate quality fail to include vitamins and minerals, whereas most nutrient profiling models fail to include whole grains. Failing to incorporate either vitamins and minerals or whole grains, the French Nutri-Score [11] penalizes calories, saturated fat, total sugar, and sodium. Energy dense grain foods, including whole grains and fortified cereals, tend to receive poor Nutri-Score ratings. However, whole grains may now be incorporated into the Australian Health Star Rating [12].

Healthy grain foods can be identified using alternative nutrient profiling methods. The published Carbohydrate Quality Indices (CQI) [13,14,15,16] are generally based on the ratios of fiber and free sugar to the carbohydrate content of food, dry weight. However, reducing the quality of grain foods to just two indices: free sugar and fiber seems overly limiting. A recent and more expansive Carbohydrate Food Quality Score (CFQS) [17,18,19] went beyond free sugar and fiber to include whole grain content, potassium and sodium.

The present objective was to rate grain foods using two parallel nutrient profiling methods. The CFQS-3 score, similar to the CQI, was based on the ratio of whole grains, free sugar, and fiber relative to carbohydrate. The Nutrient Rich Food Index (NRF9.3g) [10,20], in a new version specifically tailored to grain products, was based on percent daily values for nine key nutrients, including B vitamins and folate. The goal was to assess the contribution of healthy grains to two measures of diet quality: the Healthy Eating Index (HEI 2020) [21] and the diet- level Nutrient Rich Food (NRF) index. The consumption of healthy grains was further linked to body weight and to diabetes and metabolic health outcomes. The present hypothesis was that healthy grains, defined by the two methods, would make a significant contribution to diet quality.

## 2. Materials and Methods

### 2.1. Participant Characteristics

Dietary intakes and demographic data came from multiple cycles of the National Health and Nutrition Examination Survey (NHANES 2017–2018, 2019–2020, and 2021–2023) [22]. NHANES 2017–2020 collected data for 10,629 participants aged >0 y and NHANES 2021–2023 collected data for another 5830 participants [22,23]. The present analytical sample, limited to participants aged ≥6 y with 2 days of dietary recall, was composed of 3795 children aged 6–19 y and 10,925 adults aged 19+ y (total n = 14,720). NHANES participants were stratified by sex, age group, race/ethnicity, and family income-to-poverty ratio (IPR) level. Sex was male or female. Age groups were children (6–11 y); adolescents (12–19 y), adults (20–59 y), and older adults (>59 y). The cut-points for the family income-to-poverty ratio (IPR) were: <1; 1–1.99; 2–3.49; and ≥3.5. Race/ethnicity was defined as non-Hispanic White; non-Hispanic Black, Mexican American, other Hispanic, and other/mixed race. The National Center for Health Statistics (NCHS) Ethics Review Board (ERB) ensures that research involving human participants protects the rights and welfare of study participants and conforms to U.S. federal regulations [24].

### 2.2. Dietary Intakes Data from 24 h Recalls

The dietary component of NHANES is known as the What We Eat in America (WWEIA) study. The NHANES 24 h recall uses a multi-pass method, where respondents report the types and amounts of all foods and beverages consumed in the prior 24 h, from midnight to midnight [22]. The recall is conducted by a trained interviewer using a computerized interface. The recall first identifies a quick list of foods and beverages consumed. The time and occasion for each food item are also obtained. The second pass records the amounts consumed, followed by a final probe for any frequently forgotten foods (beverages, condiments). The present analyses were based on 2 days of dietary recalls.

### 2.3. Selection of Grain Foods

The US Department of Agriculture (USDA) Food and Nutrient Database for Dietary Studies (FNDDS) [25] provides nutrient composition data for the WWEIA studies. Data for multiple nutrients are available per 100 g, edible portion. The present data came from FNDDS 2017–2018, 2019–2020, and 2021–2023, each one associated with the corresponding NHANES cycle. When the same food code was used across multiple NHANES cycles, nutrient composition was calculated as the mean. Added sugars (tsp/100 g) were converted to g/100 g with 1 tsp equivalent to 4.2 g.

The USDA Food Patterns Equivalents Database (FPED) [26] converts 100 g of each food into cup eq or oz eq of desirable food groups in the DGA. Data for total, whole, and refined grains came from FPED 2017–2020. Based on FPED data for flour-based products (bread, cookies, bagels, biscuits), 16 g of flour equals 1 oz of grain. For intact grains (barley, bulgur, millet, oats, pasta, rice, rye, quinoa, and RTE cereals), 1 oz was 28.35 g of product.

**Figure 1:**
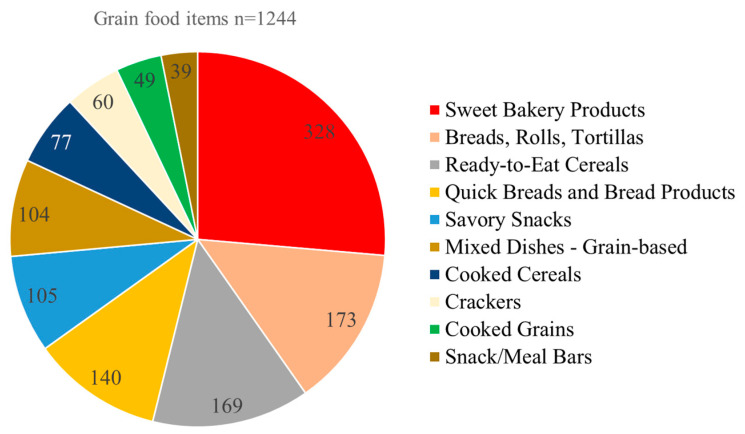
Distribution of 1244 grain foods in USDA FNDDS 2017–2018 by WWEIA food category.

Grain products were drawn from the grains, mixed dishes, and snacks and sweets food groups (one-digit WWEIA code). Two-digit WWEIA codes identified the following categories: cooked cereals (code 48), cooked grains (code 40), breads, rolls, and tortillas (code 42), quick breads and bread products (code 44), ready-to-eat cereals (code 46), savory snacks (code 50), crackers (code 52), snack/meal bars (code 54), sweet bakery products (code 55), and grain based mixed dishes (code 32). To be included, grain foods had to contain >40% of energy from carbohydrate, some total grain (>0 cup. Eq) and not contain dairy (0 cup), meat/poultry (0 cup) or seafood (0 cup). This was to eliminate mixed dishes where grain was only a minor component. The final sample was 1244 grain foods in ten categories, as seen in Figure 1.

### 2.4. Identification of Healthy Grain Foods

The quality of grain foods was assessed using two new nutrient profiling scores. The new Carbohydrate Food Quality Score CFQS-3 was a 0–3 point score. The points were awarded based on the ratios of fiber, whole grain, and added sugar to the carbohydrate content of foods. The CFQS-3 differed from previously published CQI metrics by including whole grains in addition to fiber- and sugar-to-carbohydrate ratios; foods with CFQS-3 scores ≤ 2 were classified as healthy grains. The CFQS-3 system is shown in Table 1.

The Nutrient Rich Food (NRF9.3) index is a metric of nutrient density that can be applied to individual foods and to total diets. The NRF9.3 index has two sub-scores, one based on a variable number *n* of nutrients to encourage (NRn) and the other based on 3 nutrients to limit (LIM). The food-level NRF9.3g version, tailored to grain foods, had 9 nutrients to encourage (protein, fiber, iron, niacin, thiamin, riboflavin, folate, magnesium, and vitamin E) and 3 nutrients to limit (added sugar, sodium, and saturated fat). Reference daily values (DV) came from the FDA and are shown in Table 2. All nutrient scores were calculated per 100 kcal and capped at 100%. The final NRF9.3g score was the sum of percent daily values for the sum of %DV for the 9 nutrients to encourage minus the sum of %DV for the 3 nutrients to limit. Healthy grain foods were those with NRF9.3g scores in the top tertile. The standard set of nutrient standards for the diet-level NRF9.3 is also shown in Table 2.

The amounts of total grains (g/d) and healthy grains (expressed in g/d and in % of total grains) were then calculated in diets of NHANES participants. Calculations were performed for the whole NHANES sample and by socio-demographic variables.

### 2.5. Dietary Quality Metrics NRF and HEI 2020

The classic diet-level NRF score calculated for 2000 kcal served as the second dietary quality metric. As indicated in Table 2, the 9 nutrients to encourage were protein, fiber, calcium, iron, potassium, magnesium, vitamin A, vitamin C, and vitamin D. The 3 nutrients to limit were saturated fat, added sugar and sodium. For the LIM sub-score only the excess intake of nutrients compared to the daily value was counted. In that way, diets with under 2300 mg of sodium or with under 50 g of added sugar were not penalized by the LIM score. %DV contribute to the negative score only when the maximum recommended value was exceeded. The final score was given by the sum of %DV for the 9 nutrients to encourage minus the sum of excess (in %) for the 3 nutrients to limit

The Healthy Eating Index (HEI 2020) [21] is a measure of compliance with the Dietary Guidelines for Americans (DGA) [1,2]. The HEI 2020 is a 100-point scale, composed of 13 sub-scores that include total and whole fruit, total vegetables, green vegetable and peas, dairy, whole grains, protein foods, seafoods, and plant proteins, as well as refined grains, added sugar, sodium, and saturated fat.

### 2.6. Health Outcome Measures

Health-related variables came from the NHANES clinical data files. Body mass index (BMI, kg/m^2^) was taken from NHANES examination data. BMI cut-points were based on World Health Organization (WHO) standards for adults [27] and the Centers for Disease Control and Prevention (CDC) growth charts for children (<19 y) [28]. For children cut-offs were: Underweight (BMI < 5th percentile); Normal weight (BMI 5th to <85th percentile); Overweight (BMI 85th to < 95th percentile); Obese (BMI ≥ 95th percentile). Data on waist circumference, fasting insulin and plasma cholesterol levels were for adults only (n = 10,925). Waist circumference cut-points were >102 cm in men or >88 cm in women. Insulin was measured in a fasting subsample of participants aged ≥12 y (n = 6800). Specific sample weights for this subsample from the data file were used in the analysis. The units for insulin were uU/mL and pmol/L. Total cholesterol and high-density lipoprotein (HDL) cholesterol were also for adults only. HDL-C measures were continuous and dichotomized using metabolic syndrome cut-points (<40 mg/dL in men or <50 mg/dL in women) [29].

### 2.7. The Price of Healthy Grains

The USDA Purchase to Plate Price Tool (PPPT) [30] collects scanner data from retailers and converts retail prices (USD per product) to unit prices (USD per 100 g edible portion), adjusting for consumer-level losses and waste. Prices were available for about 50% of FNNDS foods that represented 97% of total intake by weight. Mean national retail prices for 3231 food codes came from the USDA Thrifty Food Plan (TFP) supplemental data files [31]. Not all prices were available, but the TFP food prices database yielded mean prices (USD/100 g) for 786 grain foods.

### 2.8. Plan of Analyses

**Figure 2:**
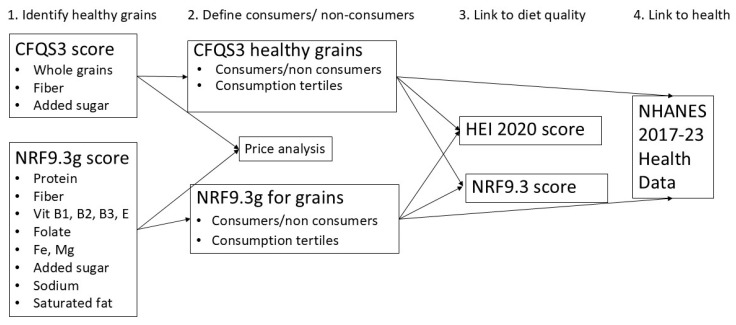
Schema design for analytical approach, identifying key steps and main variables.

Analyses used definitions of healthy grain foods based on CFQS-3 scores and on tertiles of NRF9.3g. Consumers and non-consumers of healthy grains, were characterized by socio-demographic variables and were compared on diet quality metrics, using the HEI 2020 and NRF9.3 total scores and components. Analyses of dose dependence related the amounts of healthy grains consumed (in g/day) with diet quality. All analyses accounted for the complex survey design of NHANES data. Tests of significance were conducted using chi-square for categorical variables and general linear models for continuous variables that were adjusted for covariates. Data analyses used SAS v9.4 (SAS institute, Cary, NC, USA). The analytical study design is summarized in Figure 2 below.

## 3. Results

### 3.1. Healthy Grain Foods Identified by CFQS-3

Healthy grain foods were those with most whole grains and fiber and least added sugars. Healthy grain foods scored 2 or 3 points on CFQS-3; less healthy grain foods scored 0 or 1 point. The distribution of CFQS-3 scores by category is shown in Table 3. The top three categories were cooked grains, followed by cooked cereals and savory snacks. Ready-to-eat cereals were next, largely satisfying the whole grain criterion (71%); however, this category mostly failed on added sugar. Bread came next, with low added sugar scores. Most grain based mixed dishes scored low, except on added sugar. At the bottom, with zero scores, were sweet bakery products, snack/meal bars, and quick breads.

Cooked grains was the only category where most foods had CFQS-3 scores of ≥2. All other categories had <25% of foods with zero scores and more foods with scores of 2 or 3 (between 33% and 55%).

### 3.2. Healthy Grain Foods Identified by NRF9.3g

Table 4 shows that the NRF9.3g scores ranged from −28 to 608 with a mean of 40.9. RTE cereals, cooked cereals, and breads/rolls/tortillas had the highest scores, mostly in the top tertile of NRF9.3g. Fortified RTE cereals and fortified high protein snack meal bars also received very high scores. Crackers, cooked grains, and grain based mixed dishes were mostly in the second tertile. Savory snacks, most snack/meal bars, and quick breads got lower NRF9.3g scores. Sweet bakery products got low NRF9.3g scores that were mostly (89%) in the bottom tertile. Differences in NRF9.3g scores by category were statistically significant (*p* < 0.001) by one-way analysis of variance (ANOVA). Tertile 1 and tertile 2 cut-points of NRF9.3g among the 1244 grain foods were −17.5/100 kcal and 45.8%/100 kcal, respectively.

### 3.3. Healthy Grain Foods Consumption by Socio-Demographics

Mean consumption of grain foods in this NHANES sample was 160 g/d (Table 5). Men consumed more grain foods than did women (174 g/d vs. 147 g/d). There was no significant effect of age group or IPR. The Other race/ethnicity group consumed much more grains (214 g/d) than did the rest of the groups (from 149 to 162 g/d).

The proportion of healthy grains in the diet (as percent of total grains) was the most relevant metric. First, there were strong effects of age group and income. Older adults and higher income groups consumed a higher proportion of whole grains. The non-Hispanic Black group had the lowest proportion of healthy grains.

Incomes did not affect the consumption of healthy grains defined using the NRF9.3g metric. Distribution by age was bimodal, with lowest proportion of healthy grains observed among 20–60 y olds (31% of total grains). The Non-Hispanic White group consumed the highest proportion of healthy grains (36% of total grains).

Table 6 shows further socio-demographic analyses of healthy grain foods as identified by the CFQS-3. Two alternative metrics are presented: a comparison of consumers versus non consumers (left columns) and distribution of tertiles of healthy grain consumption (right columns). Here, foods identified as healthy grains were those that contained whole grains and fiber and were low in added sugar.

More likely to consume healthy grains were women, older adults, higher income groups, and Mexican Americans. More likely to fall in the top tertile of the amounts of healthy grains consumed were women, older adults, higher income groups and Mexican Americans. The effects of age and income were particularly strong.

Table 7 shows analyses of healthy grain consumption, identified by NRF9.3g, by socio-demographics. Two alternative metrics are presented: a comparison of consumers versus non consumers (left columns) and distribution of tertiles of healthy grain consumption (right columns). Here, healthy grains are grain foods with high content of protein, fiber, vitamins, and minerals, and low content of saturated fat, added sugar, and sodium.

The effects of age were bimodal, with teenagers and older adults more likely to consume higher amounts of healthy grains established with NRF9.3g. There was also a weak effect of income. The non-Hispanic White group was most likely to consume healthy grains; the non-Hispanic Black group was least likely. More likely to fall in the top tertile of the amounts of healthy grains consumed was the non-Hispanic White group.

### 3.4. Healthy Grains and Diet Quality Metrics: The HEI 2020

**Figure 3:**
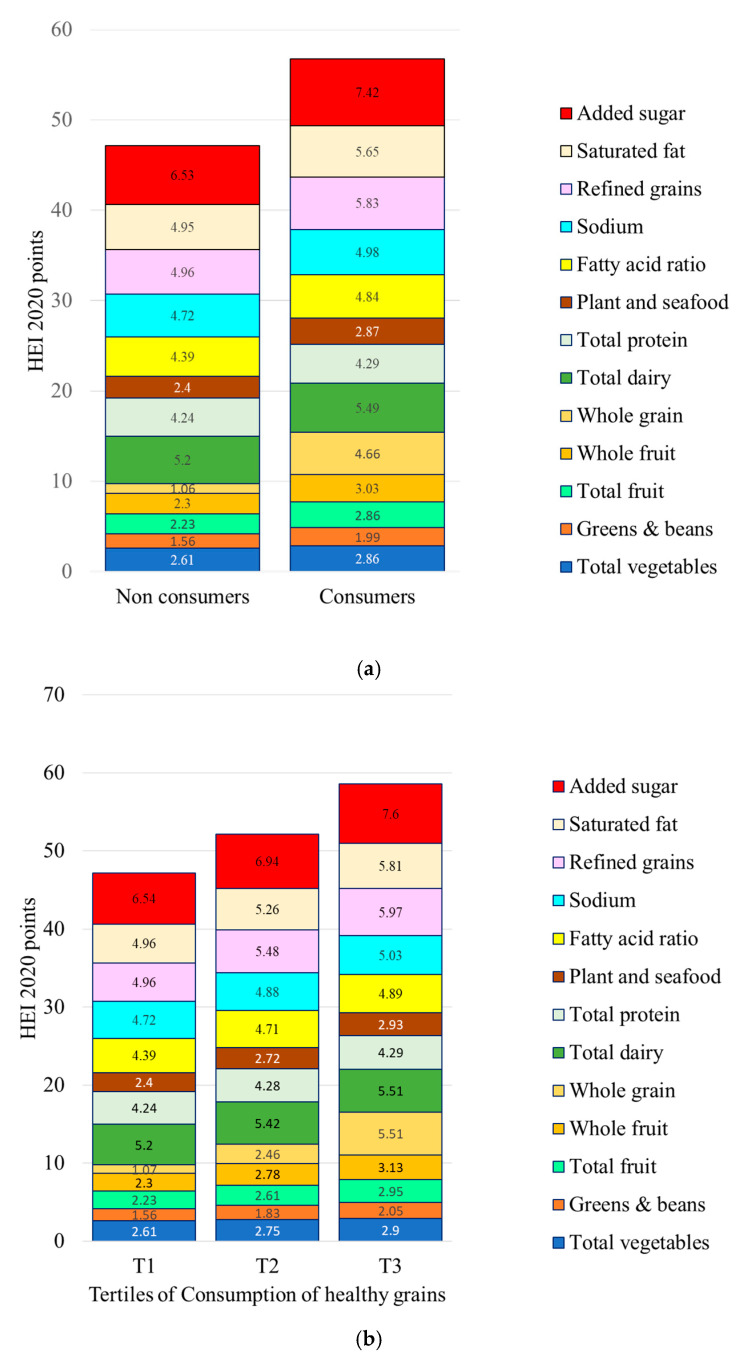
Healthy Eating Index (HEI 2020) total scores and sub-scores for consumers (C) and non-consumers (NC) of healthy grains (**a**) and by tertile of consumption (**b**) of healthy grains identified by CFQS-3. Means are adjusted for energy intakes, age, gender, ethnicity, and IPR.

Figure 3 shows the relation between healthy grain consumption and HEI 2020 scores. First, consumers of healthy grains identified with CFQS-3 had significantly higher HEI 2020 total scores and selected sub-scores. There were significant differences in whole grains (more), added sugar (less), refined grains (less), and saturated fat (less). Consumers of healthy grains also consumed more fruit. These effects were dose-dependent, with higher consumption of healthy grains associated with progressively higher HEI 2020 scores (Figure 3b). Data for Figure 3 are provided in Supplementary Table S3.

**Figure 4:**
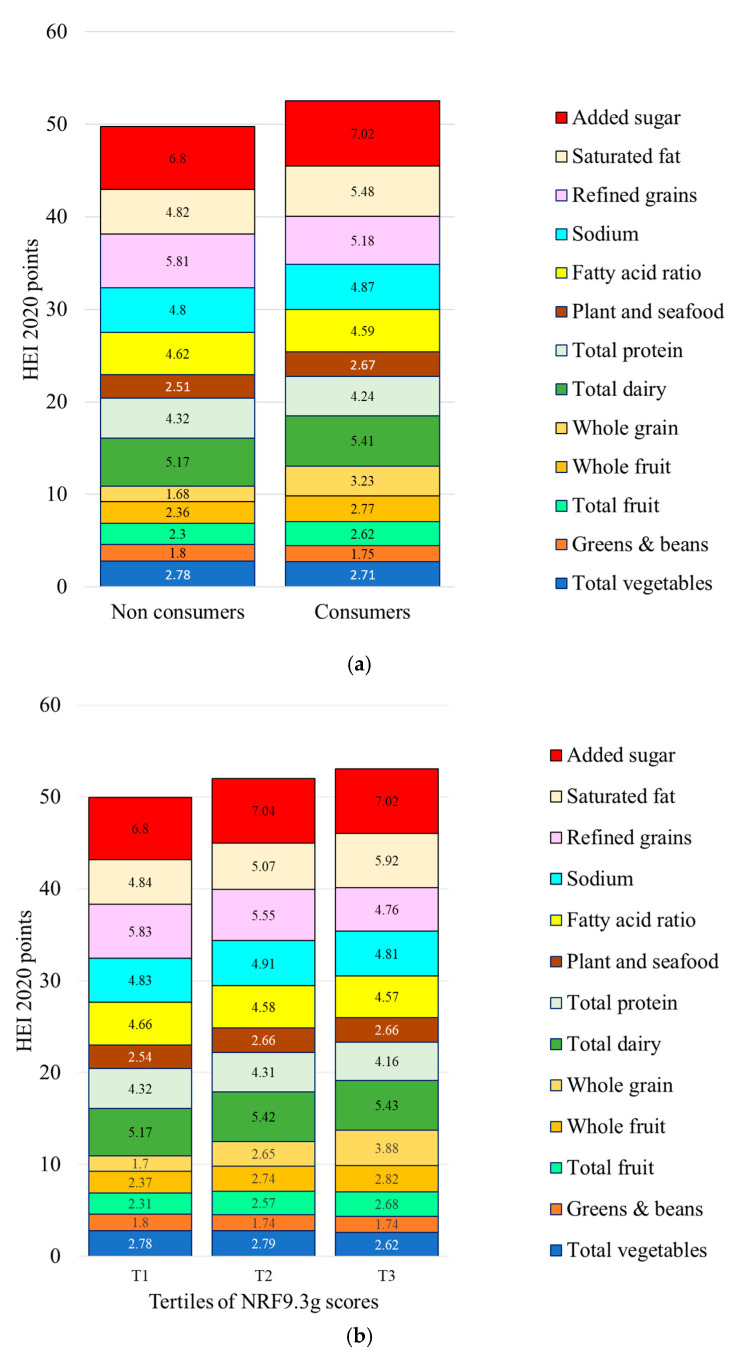
Healthy Eating Index (HEI 2020) total scores and sub-scores for consumers (C) and non-consumers (NC) of healthy grains (**a**) and by tertile of consumption (**b**) of healthy grains identified by NRF9.3g. Means are adjusted for energy intakes, age, gender, ethnicity, and IPR.

The relation between healthy grains identified using NRF9.3g and HEI 2020 scores is shown in Figure 4. Consumers of healthy grains had higher HEI 2020 total scores and selected sub-scores. There were significant differences in whole grains (more), added sugar (less), refined grains (less), and saturated fat (less). Consumers of healthy grains also consumed more fruit. Data are provided in Supplementary Table S4.

### 3.5. Healthy Grains and Diet Quality Metrics: Diet-Level NRF Score

**Figure 5:**
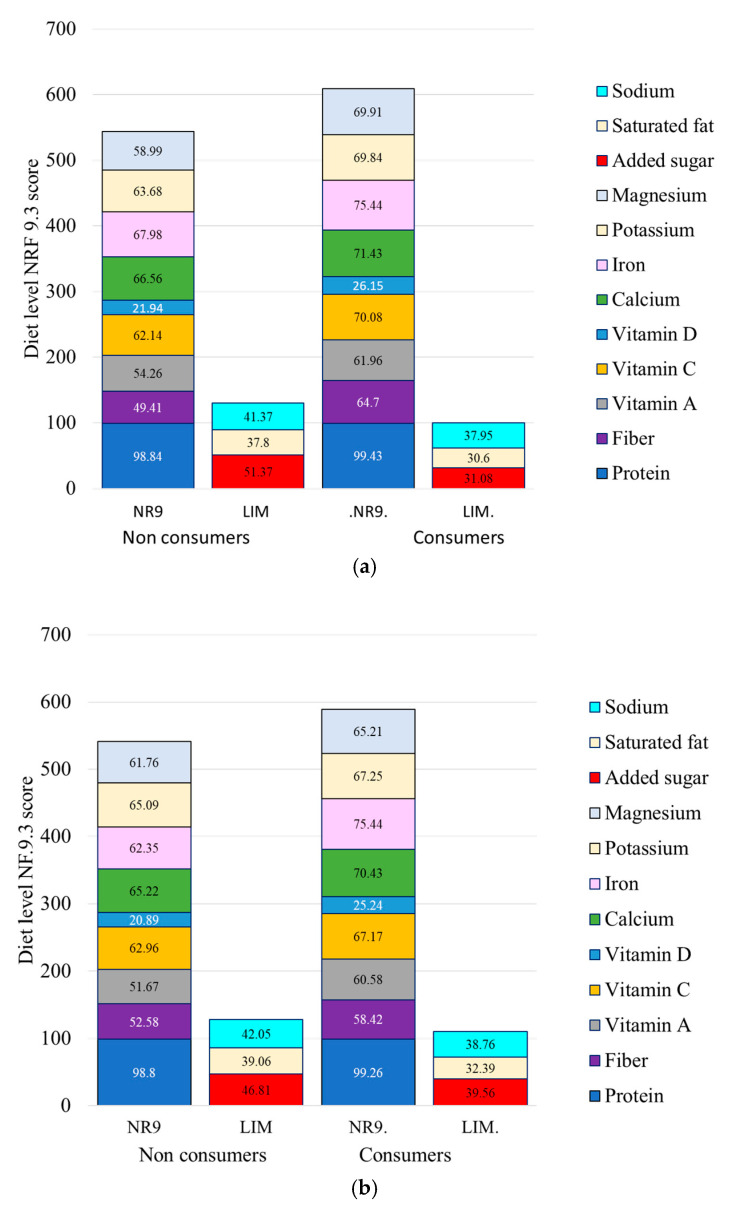
Diet-level NRF9.3 nutrient density scores and components for consumers and non-consumers of healthy grains identified using CFQS-3 (**a**) and NRF9.3g (**b**). NR9 is the positive sub-score of the diet-level NRF scoring system, whereas LIM is the negative sub-score. Means are adjusted for energy intakes, age, gender, ethnicity, and IPR.

Figure 5 shows that consumers of healthy grains identified with CFQS-3 had significantly higher diet-level NRF total scores and selected sub-scores. There were significant increments in protein, fiber, calcium, iron, potassium, and magnesium. Consumers of healthy grains also consumed less added sugar, sodium, and saturated fat. These effects were weakly dose-dependent, with higher consumption of healthy grains associated with progressively higher NRF scores. Data for Figure 5 are in Supplementary Table S4.

### 3.6. The Price of Healthy Grain Foods

Healthy grain foods identified using CFQS-3 cost less per 100 g compared to less healthy grain foods (Table 8). There was no difference in cost per 100 kcal. Healthy grains identified using NRF9.3g cost less per 100 g and per 100 kcal than did less healthy grain foods. Given that the NRF9.3g metric is sensitive to fortification, these data suggest that nutrient rich (fortified) grain foods can be obtained at an affordable cost. The data, based on USDA national food prices, show that healthy grain foods are not necessarily more expensive per calorie or per gram.

### 3.7. Analysis of Cross Sectional Health Outcomes

Data on body weight, waist circumference, fasting insulin, and plasma cholesterol levels (total and HDL) were for adults only. Insulin levels were measured in smaller subsample of 6800 participants. Consumers and non-consumers of healthy grains were compared adjusting for energy intakes, age, gender, ethnicity, and IPR (Table 9).

Consumers of healthy grains were less likely to live with obesity. Consumers of healthy grains had significantly lower fasting plasma insulin levels (13.97 vs. 15.90 uU/mL; *p* < 0.001). No differences were obtained for waist circumference or for total cholesterol. There was a weak association with higher HDL levels for consumers of healthy grains identified using the CFQS-3 method.

## 4. Discussion

### 4.1. Healthy Grain Foods and Consumption Patterns

The present analyses used two new methods to identify healthy grain foods. The first method, the CFQS-3 was based on the product content of whole grains, fiber, and added sugars relative to carbohydrate. That particular system resembled previously published Carbohydrate Quality Indices or CQI [13,14,15], but with the addition of whole grains. The CFQS-3 scoring system favored cooked grains, cooked cereals, mixed grain dishes, and savory snacks. Sweet bakery goods containing refined grains, added sugar, and little fiber got zero points. These data are consistent with earlier studies [18,19] that applied another version of the CFQS (CFAQ-5) 1561 grain foods in the FNDDS 2017–2018 nutrient composition database. Grain foods that received highest scores were whole grain cooked grains and cereals, as well as whole grain pasta, crackers, and breads

The new NRF9.3g score was tailored specifically to grain foods. The nine nutrients to encourage that are commonly associated with flour and grain foods were protein, fiber, thiamin, niacin, riboflavin, folate, iron, and vitamin E. Since the NRF approach was mostly nutrient-based, fortified products received high scores. The highest scores were given to ready-to-eat (RTE) breakfast cereals, cooked grains, and breads and rolls.

Given the DGA admonition to make at least half the grains whole [1], it is worth noting that the percentage of healthy grain (as percent of total) did not approach anywhere near 50% of all grain foods either in the total sample or in any population subgroup. Depending on the method used, the percentage of healthy grains in the diet varied between 19% and 33%. Older adults and Mexican Americans came closest with 25% of total grain foods consumed classified as healthy.

More likely to consume healthy grains identified by CFQS-3 were women, higher income groups, and Mexican Americans. Those results are consistent with prior findings of a social gradient in whole grain consumption [32,33]. In past studies, consumption of whole grains was associated with higher socioeconomic status, education, and incomes [34]. By contrast, the consumption of RTE cereals was not associated with higher incomes [9]. Consistent with that report, we found no association between healthy grains identified using NRF9.3g nutrient density score and IPR. Significant differences were obtained for age groups and for race/ethnicity. More likely to consume healthy grains were younger and older adults and non-Hispanic White individuals. Non-Hispanic Black individuals were least likely to consume healthy grains.

It is worth noting that the healthier grains, identified using both methods, were not more expensive. Mean USDA 2021 food prices were not significantly different. Healthy grains identified using NRF9.3g with its emphasis on fortification were actually cheaper. These analyses indicate that price may not be a barrier to adopting diets with healthy grains, whether whole grain cereals or fortified RTE cereals [9].

### 4.2. Healthy Grains Consumption, Diet Quality and Selected Health Outcomes

Consumption of healthy grain foods, identified using CFQS-3 and the NRF9.3g methods, was associated with higher HEI 2020 scores and selected sub-scores, indicating better compliance with the 2020–2025 DGA. Analysis of HEI 2020 sub-scores showed that consumers of healthy grain foods also consumed less added sugar, sodium, and saturated fat. Significant differences were observed for other sub-scores, notably refined grains (less) and whole grains (more). The effect was dose-dependent, with higher amounts of healthy grains associated with progressively higher HEI 2020 scores. Past studies in this area have also pointed to associations between higher carbohydrate quality and higher quality of the total diet, most often calculated using HEI scores. However, carbohydrate quality tended to be defined in terms of whole grains, fiber, or the glycemic index of foods [35,36].

In this study, consumption of healthy grain foods, identified by both methods, was also associated with higher dietary nutrient density. Consumers had diets that were higher in dietary protein, fiber, calcium, iron, potassium, and magnesium. This effect was also dose-dependent.

The cross sectional NHANES clinical data provide some insights into the health of consumers and non-consumers of healthy grains. In the present analyses, consumers of healthy grain foods were less likely to be living with obesity and had lower fasting insulin levels. Conversely, consumers of less healthy grains (sweet bakery goods) were more likely to fall into the obese category.

Past studies on diets and health have also linked carbohydrate quality with improved health outcomes [37,38]. Again, the carbohydrate quality metrics were largely based on whole grains and fiber. Studies have linked whole grain consumption with lower BMI values in children [39] and adults [40], reduced adiposity [41], and lower fasting insulin levels [42]. Whole grain consumption has been linked to reduced risk of cardiovascular disease, cancer, and all-cause mortality [43]. The Global Burden of Disease studies have also identified whole grains as a key dietary factor in disease prevention [44]. The present analyses identified healthy grains based not only on their whole grain content but on a more diverse composite nutrient profile.

### 4.3. Healthy Grain Foods in the NOVA Classification System

The recent MAHA Report [45] has introduced a new distinction between “ultra-processed grains” and the healthier nutrient-rich whole grains. It is worth noting that the nutrient rich whole grain breads also fall into the ultra-processed category, based on the current NOVA classification system [46]. Grain foods are seldom eaten raw, and grains need to be processed in some way [47]. The two nutrient profiling models presented in the paper bypass the “ultra-processed” controversy and simply identify healthy grain foods based on nutrients and dietary ingredients. The highest scores are given to items that contain whole grains and fiber, are low in added sugars, and/or are fortified with vitamins and minerals. Other researchers have also explored carbohydrate quality of packaged carbohydrate foods based on nutrients and ingredients [33,34].

### 4.4. Study Limitations

The study had limitations. NHANES 24h dietary recalls are self-reports that are subject to misreporting and recall bias. The definition of whole grains was wholly dependent on the USDA FPED file. Nutrient profiling models provide a way to quantify nutrient density of foods; however, the final score may depend on the nutrients selected, standards used and the assumptions made. For that reason, nutrient profiles are only “models” of nutrient density or the healthfulness of foods. Cross-sectional study design of NHANES precludes any considerations of causality; any links to health variables need to be viewed only as associations. Residual confounding may have influenced associations due to dietary restraint, physical activity, or other unmeasured variables.

## 5. Conclusions

Healthy grain foods contain whole grains and fiber, limit added sugars, and contain vitamins and minerals, notably B vitamins and folic acid. The list ranges from cooked grains, whole wheat bread, crackers, cereals, and pasta to lower-sugar fortified RTE cereals and cereal bars. These multiple aspects of healthy grains are difficult to capture using a single nutrient profiling system. The present findings, based on two separate nutrient profiling systems, show that healthy grains were associated with healthier diets and selected metabolic health outcomes.

## Figures and Tables

**Table 1:** Carbohydrate Food Quality Score (CFQS-3) components and selection criteria.

CFQS-3	Component Scores	Score Range
Fiber	1 point if fiber >10 g/100 g carb portion; else 0 point	0 to 1
Added sugars	1 point if added sugar <10 g/100 g carb portion; else 0 point	0 to 1
Whole grains	1 point if whole grains >25 g/100 g dry weight; else 0 point	0 to 1
CFQS-3	Sum of points	0 to 3

**Table 2:** Reference daily values used for food-level and diet-level NRF9.3.

NRF9.3g Identifies Healthy Grain Foods		NRF Identifies Healthy Diets	
Nutrients	Reference Daily Values	Nutrients	Reference Daily Values
Protein	50 g	Protein	50 g
Fiber	28 g	Fiber	28 g
Iron	18 mg	Vitamin A	900 mcg RAE
Vitamin B1 (thiamin)	1.2 mg	Vitamin C	90 mg
Vitamin B2 (riboflavin)	1.3 mg	Iron	18 mg
Vitamin B3 (niacin)	16 mg	Potassium	4700 mg
Folate	400 mcg	Magnesium	420 mg
Magnesium	420 mg	Calcium	1300 mg
Vitamin E	15 mg	Vitamin D	20 mcg
Added sugar	50 g	Added sugar	50 g
Saturated fatty acid	20 g	Saturated fatty acid	20 g
Sodium	2300 mg	Sodium	

**Table 3:** Percent of grain foods that scored points on free sugar, whole grains, and fiber CFQS-3 sub-scores by category. Table is color coded as a heatmap (green: >40%; red: <30%; orange else).

Grain Categories	N	Fiber ^1^	Added Sugar ^2^	Whole Grain ^3^
All Grain Foods	1244	21.4	43.8	30.7
Cooked Cereals	77	42.8	66.2	54.5
Cooked Grains	49	30.6	97.9	44.8
Savory Snacks	105	39.0	86.6	49.5
Ready-to-Eat Cereals	169	40.2	12.4	71.0
Breads, Rolls, Tortillas	173	30.6	72.8	33.5
Crackers	60	30.0	63.3	31.6
Mixed Dishes—Grain-based	104	12.5	96.1	29.8
Quick Breads and Bread Products	140	6.4	36.4	20.7
Snack/Meal Bars	39	23.0	0.0	10.2
Sweet Bakery Products	328	2.4	6.0	1.5

^1^ fiber criteria: >10 g/100 g carbohydrate; ^2^ added sugar criteria: <10 g/100 g carbohydrate; ^3^ whole grain criteria: >25 g/100 g dry weight.

**Table 4:** Means (SD, min, max) of NRF9.3g scores for all grain foods and by category.

Grain Categories	N	Mean	SD	Min	Max
All grain foods	1244	40.9	51.2	−28.0	608.7
Ready-to-Eat Cereals	169	128.7	76.0	25.8	608.7
Cooked Cereals	77	52.9	27.4	0.5	144.2
Breads, Rolls, Tortillas	173	49.5	11.8	15.5	80.9
Snack/Meal Bars	39	48.7	59.4	−19.5	283.8
Mixed Dishes—Grain-based	104	40.1	15.0	−23.8	77.5
Cooked Grains	49	33.8	13.1	0.3	60.7
Crackers	60	30.5	11.1	8.4	61.9
Quick Breads and Bread Products	140	29.1	19.7	−1.6	87.7
Savory Snacks	105	21.2	16.1	−26.8	60.4
Sweet Bakery Products	328	2.0	12.7	−28.0	47.7

**Table 5:** Mean amount of total grains (g/d) and amount of healthy grains (in g/d and % of total grains) established with CFQS-3 or tertiles of NRF9.3g.

	Total Grain Foods				Healthy Grains Defined by CFQS-3						Healthy Grains Defined by NRF9.3g					
	g/d				g/d			% of Total Grains			g/d			% of Total Grains		
	N	Mean	SD	*p* *	Mean	SD	*p*	Mean	SD	*p*	Mean	SD	*p*	Mean	SD	*p*
All	14,720	160.45	139.58		35.78	76.64		19.09	28.57		53.13	76.72		33.02	33.28	
Income-to-poverty ratio																
0–0.99	2583	149.38	145.44	0.0702	28.79	70.47	0.0004	16.39	28.10	0.0025	50.51	84.70	0.1337	33.26	35.03	0.1251
1–1.99	3140	156.26	146.13		31.89	72.40		17.67	28.66		53.55	81.19		34.25	34.38	
2–3.49	2941	159.01	128.87		31.25	66.17		17.83	27.39		51.88	72.40		32.49	32.80	
3.50–5	4394	167.37	137.29		41.41	85.10		20.78	28.81		55.87	75.61		33.49	32.40	
Sex																
Male	6927	174.27	158.02	<0.0001	38.08	86.81	0.0071	18.17	28.54	0.0351	56.78	85.00	<0.0001	32.52	33.42	0.1608
Female	7793	147.31	117.93		33.60	65.43		19.97	28.58		49.65	67.73		33.50	33.13	
Age classes																
6–11 y	1678	165.57	104.21	0.0752	24.90	45.77	<0.0001	14.28	21.68	<0.0001	57.09	62.90	<0.0001	34.56	28.71	<0.0001
12–19 y	2117	159.02	149.51		24.36	56.67		14.37	25.06		57.27	92.78		34.43	33.95	
20–59 y	6317	156.81	145.62		34.53	77.35		18.64	29.03		49.24	75.86		30.97	33.90	
≥60 y	4608	167.78	130.28		47.63	88.79		23.94	30.31		58.80	74.08		36.57	32.57	
Ethnicity																
Mexican American	1576	149.51	158.02	<0.0001	41.52	86.81	<0.0001	24.90	28.54	<0.0001	41.26	85.00	<0.0001	28.74	33.42	<0.0001
Non-Hispanic Black	3271	148.13	134.56		24.58	62.09		14.23	26.38		44.54	69.54		30.15	34.10	
Non-Hispanic White	6345	154.56	129.35		34.69	74.62		19.02	28.43		57.53	79.37		35.97	33.79	
Other Hispanic	1447	162.30	117.93		33.43	65.43		18.27	28.58		45.54	67.73		27.64	33.13	
Other Race-Including Multi-Racial	2081	214.63	188.79		51.12	98.94		20.52	28.83		54.31	82.33		27.79	31.26	

* Statistics based on Chi square. *p*-values indicate significant differences across socio-demographic variables.

**Table 6:** Distribution of NHANES participants by socio-demographic variables (income-to-poverty ratio, sex, age classes, and ethnicity) in the whole sample and among non-consumers and consumers of healthy grains identified by CFQS-3 method. Data show column percentages and statistics.

	Non-Consumers and Consumers of Healthy Grains (CFQ-3 Based)				Tertiles of Consumptions of Healthy Grains (CFQS-3 Based)				
	NC *	C *	Total		T1 ** [0,0] ***	T2 [0,225]	T3 [225,1400]	Total	
	%	%	%	*p*	%	%	%	%	*p*
IPR				<0.0001					<0.0001
0–0.99	14.85	10.75	12.93		14.85	11.60	10.43	12.93	
1–1.99	18.53	15.31	17.03		18.53	14.90	15.47	17.03	
2–3.49	20.76	19.96	20.38		20.76	22.35	19.07	20.38	
3.50–5	35.70	43.15	39.17		35.70	40.19	44.26	39.17	
Sex									
Male	51.84	45.21	48.75	<0.0001	51.84	37.94	47.92	48.75	<0.0001
Female	48.16	54.79	51.25		48.16	62.06	52.08	51.25	
Age classes									
6–11 y	7.75	8.65	8.17	<0.0001	7.75	12.75	7.12	8.17	<0.0001
12–19 y	12.97	9.56	11.38		12.97	10.08	9.36	11.38	
20–59 y	59.76	51.85	56.07		59.76	48.61	53.06	56.07	
≥60 y	19.52	29.95	24.38		19.52	28.56	30.47	24.38	
Ethnicity									
Mexican American	7.55	11.00	9.16	<0.0001	7.55	8.95	11.76	9.16	<0.0001
Non-Hispanic Black	14.51	8.53	11.72		14.51	10.16	7.92	11.72	
Non-Hispanic White	58.67	60.84	59.68		58.67	63.50	59.85	59.68	
Other Hispanic	9.33	8.06	8.73		9.33	7.97	8.09	8.73	
Other/Multi-Racial	9.94	11.58	10.70		9.94	9.41	12.38	10.70	

* NC: non-consumers; C: consumers; ** T1, T2, T3 tertiles, *** tertile cut-points in grams. Statistics based on Chi square. *p*-values indicate significant differences across socio-demographic variables.

**Table 7:** Distribution of NHANES participants by socio-demographic variables (income-to-poverty ratio, sex, age classes and ethnicity) in the whole sample and among non-consumers and consumers of healthy grains identified by CFQS-3 method. Data by consumption tertiles are also provided. Data shows column percentages and statistics.

	Non-Consumers and Consumers of Healthy Grains (NRF9.3g Based)				Tertiles of Consumptions of Healthy Grains (NRF9.3g Based)				
	NC *	C *	Total	*p*	T1 ** [0,105] ***	T2 [105,525]	T3 [525,1330]	Total	*p*
IPR	%		%		%	%	%	%	
0–0.99	14.45	12.25	12.93	0.0012	14.56	12.26	12.00	12.93	0.0269
1–1.99	17.43	16.85	17.03		17.19	16.98	16.92	17.03	
2–3.49	20.54	20.31	20.38		20.41	20.24	20.50	20.38	
3.50–5	35.58	40.80	39.17		36.06	40.53	40.90	39.17	
Sex									
Male	50.41	48.00	48.75	0.0158	50.10	44.75	51.34	48.75	<0.0001
Female	49.59	52.00	51.25		49.90	55.25	48.66	51.25	
Age classes									
6–11 y	5.04	9.59	8.17	<0.0001	5.45	9.31	9.72	8.17	<0.0001
12–19 y	11.54	11.30	11.38		11.48	10.51	12.12	11.38	
20–59 y	65.42	51.83	56.07		65.10	53.26	49.97	56.07	
≥60 y	17.99	27.28	24.38		17.96	26.93	28.19	24.38	
Ethnicity									
Mexican American	9.87	8.84	9.16	<0.0001	9.83	10.63	7.07	9.16	<0.0001
Non-Hispanic Black	14.55	10.43	11.72		14.39	10.54	10.26	11.72	
Non-Hispanic White	54.27	62.13	59.68		53.97	60.16	64.81	59.68	
Other Hispanic	9.87	8.22	8.73		10.31	8.66	7.26	8.73	
Other/Multi-Racial	11.43	10.38	10.70		11.50	10.02	10.59	10.70	

* NC: non-consumers; C: consumers; ** T1, T2, T3 tertiles, *** tertile cut-points. Statistics based on Chi square. *p*-values indicate significant differences across socio-demographic variables.

**Table 8:** Mean price (in USD per 100 g and per 100 kcal) in a subsample of grain foods (n = 786) by nutrient density scores: CFQS-3 (0, 1, 2 or 3) or tertiles of NRF9.3g.

		Price, $/100 g				Price, $/100 kcal			
	N	Mean ^2^	SD	95% LCL	95% UCL	Mean ^2^	SD	95% LCL	95% UCL
Sub sample ^1^	786	0.89	0.51	0.86	0.93	0.27	0.16	0.26	0.28
CFQS-3									
0 point	334	0.95 ^a^	0.40	0.91	1.00	0.27 ^a^	0.12	0.26	0.28
1 point	259	0.83 ^b^	0.61	0.75	0.90	0.26 ^a^	0.19	0.24	0.29
2 or 3 points	193	0.87 ^a,b^	0.50	0.80	0.94	0.27 ^a^	0.18	0.25	0.30
*p*		0.0100				0.8337			
NRF9.3g									
T1	281	1.03 ^a^	0.42	0.98	1.08	0.28 ^a^	0.13	0.27	0.30
T2	231	0.93 ^a^	0.68	0.85	1.02	0.29 ^a^	0.22	0.26	0.31
T3	274	0.71 ^b^	0.33	0.67	0.75	0.24 ^b^	0.12	0.23	0.26
*p*		<0.001				0.0017			

^1^ 2021 prices. Prices were not available for entire sample of grain foods. ^2^ Means were statistically compared with a GLM test. Two-by-two comparisons were Bonferroni adjusted. Two means sharing the same letter indicate a non-significant difference.

**Table 9:** Anthropometric and selected clinical data by consumption of healthy grains identified using CFQS-3 and NRF9.3g methods. Body weight for all participants (n = 14,720). Waist circumference and HDL-C data for adults only (n = 10,925).

	CFQ-3 Based				NRF9.3 Based			
	NC *	C *	All	*p*	NC *	C *	All	*p*
	%	%	%		%	%	%	
Body Weight								
Underweight	1.72	2.08	1.89	<0.0001	1.60	2.02	1.89	<0.0001
Normal weight	29.38	32.27	30.72		26.91	32.45	30.72	
Overweight	27.16	30.31	28.63		29.02	28.46	28.63	
Obese	41.09	34.56	38.04		41.85	36.32	38.04	
Missing	0.65	0.78	0.71		0.62	0.75	0.71	
Waist circumference								
Below	38.27	40.29	39.22	0.3457	39.47	39.11	39.22	0.9649
Above (Obese)	59.13	57.34	58.28		58.03	58.40	58.28	
Missing	2.61	2.38	2.50		2.51	2.49	2.50	
HDL cholesterol								
Low	27.42	23.99	25.80	0.0482	25.04	26.16	25.80	0.1348
High	66.01	69.87	67.84		67.40	68.05	67.84	
Missing	6.57	6.14	6.36		7.55	5.80	6.36	

* NC: non consumers, C: consumers. Statistics for BMI categories based on Chi square. *p*-values indicate significant differences across socio-demographic variables.

## Data Availability

Publicly available datasets were analyzed in this study. This data can be found at: https://wwwn.cdc.gov/nchs/nhanes/analyticguidelines.aspx (accessed on 12 June 2025); https://catalog.data.gov/dataset/food-and-nutrient-database-for-dietary-studies-fndds-f9910 (accessed on 15 August 2024); http://ars.usda.gov/northeast-area/beltsville-md-bhnrc/beltsville-human-nutrition-research-center/food-surveys-research-group/docs/fped-databases/ (accessed on 8 April 2025); https://fns-prod.azureedge.us/sites/default/files/resource-files/TFP2021.pdf (accessed on 5 August 2025). The original contributions presented in the study are included in the article; further inquiries can be directed to the corresponding author.

## Supplementary Materials

The following supporting information can be downloaded at: https://www.mdpi.com/article/10.3390/nu17162674/s1, Table S1. Comparison of raw mean diet quality scores (HEI and NRF) between consumers and non-consumers of healthy grains and between tertiles of consumption established with CFQS3, Table S2. Comparison of raw mean diet quality scores (HEI and NRF) between consumers and non-consumers of healthy grains and between tertiles of consumption established with NRF9.3g, Table S3. Comparison of adjusted meandiet quality scores (HEI and NRF) between consumers and non-consumers of healthy grains and between tertiles of consumption established with CFQS3, Table S4. Comparison of adjusted mean diet quality scores (HEI and NRF) between consumers and non-consumers of healthy grains and between tertiles of consumption established with NRF9.3g.
