# Supplementary material for: Healthy Grains in Healthy Diets: The Contribution of Grain Foods to Diet Quality and Health in the National Health and Nutrition Examination Survey 2017–2023
Source: Nutrients. 2025 Aug 19;17(16):2674. doi: 10.3390/nu17162674 (PMC12389150; doi:10.3390/nu17162674)
Supplement: Supplementary file 1 [file nutrients-17-02674-s001.zip › nutrients-3792667-supplementary.pdf]

SUPPLEMENTARY TABLES

Supplementary Table S1. Comparison of nutritional scores (HEI and NRF) between consumers and non-consumers of healthy grains and between tertiles of consumption established with CFQS3. Raw scores and p values.

	Non-consumers and consumers of health grains (CFQ3 based)					Tertiles of consumptions of healthy grains (CFQS3 based)						
	NC		C			T1 (0g/d)		T2 (<=225g/d)		T3 (>225g/d)		
	Mean	SE	Mean	SE	P	Mean	SE	Mean	SE	Mean	SE	P
**0_DRXTKCAL**	2005.63	10.99	2057.97	14.44	0.0057	2005.63	10.99	1894.50	26.97	2118.92	16.88	<.0001
**10_NRF9**	403.89	4.47	509.11	4.03	<.0001	403.89	4.47	468.15	6.09	524.38	4.41	<.0001
**11_NR**	540.98	2.79	612.62	2.92	<.0001	540.98	2.79	585.26	4.65	622.82	3.48	<.0001
**12_ratio_p_prot**	98.89	0.09	99.52	0.05	<.0001	98.89	0.09	99.42	0.11	99.55	0.06	<.0001
**13_ratio_p_fibre**	48.44	0.47	64.85	0.51	<.0001	48.44	0.47	56.65	0.69	67.91	0.58	<.0001
**14_ratio_p_vitA**	55.19	0.67	64.61	0.71	<.0001	55.19	0.67	63.62	1.11	64.98	0.83	<.0001
**15_ratio_p_vitC**	58.55	0.78	68.05	0.73	<.0001	58.55	0.78	65.69	1.37	68.93	0.75	<.0001
**16_ratio_p_vitD**	20.15	0.29	24.50	0.46	<.0001	20.15	0.29	23.50	0.58	24.88	0.57	<.0001
**17_ratio_p_calcium**	66.02	0.48	71.00	0.51	<.0001	66.02	0.48	69.57	0.71	71.53	0.64	<.0001
**18_ratio_p_iron**	66.86	0.32	74.39	0.42	<.0001	66.86	0.32	70.12	0.42	75.98	0.52	<.0001
**19_ratio_p_potassium**	65.46	0.45	72.65	0.39	<.0001	65.46	0.45	69.67	0.88	73.77	0.46	<.0001
**20_ratio_p_magnesium**	61.43	0.38	73.04	0.37	<.0001	61.43	0.38	67.02	0.73	75.28	0.41	<.0001
**21_LIM**	137.10	2.12	103.51	1.79	<.0001	137.10	2.12	117.12	2.39	98.44	1.80	<.0001
**22_ratio_n_add_sugar**	53.82	1.91	30.69	1.39	<.0001	53.82	1.91	40.07	2.44	27.19	1.28	<.0001
**23_ratio_n_sfa**	39.21	0.82	33.25	0.86	<.0001	39.21	0.82	36.15	1.04	32.17	0.91	<.0001
**24_ratio_n_sodium**	44.07	0.78	39.57	0.57	<.0001	44.07	0.78	40.89	1.37	39.08	0.64	<.0001
**25_HEI2015_TOTAL_SCORE**	47.47	0.38	57.80	0.40	<.0001	47.47	0.38	53.10	0.54	59.55	0.46	<.0001
**26_HEI2015C1_TOTALVEG**	2.82	0.03	3.16	0.03	<.0001	2.82	0.03	3.03	0.07	3.20	0.04	<.0001
**27_HEI2015C2_GREEN_AND_B**	1.59	0.06	2.11	0.06	<.0001	1.59	0.06	1.89	0.10	2.19	0.06	<.0001
**28_HEI2015C3_TOTALFRUIT**	1.92	0.05	2.65	0.05	<.0001	1.92	0.05	2.45	0.08	2.72	0.05	<.0001
**29_HEI2015C4_WHOLEFRUIT**	2.08	0.05	2.98	0.06	<.0001	2.08	0.05	2.76	0.08	3.07	0.06	<.0001
**30_HEI2015C5_WHOLEGRAIN**	1.00	0.03	4.57	0.07	<.0001	1.00	0.03	2.47	0.08	5.35	0.10	<.0001

**31_HEI2015C6_TOTALDAIRY**	5.01	0.05	5.34	0.07	0.0003	5.01	0.05	5.36	0.11	5.34	0.09	0.0009
**32_HEI2015C7_TOTPROT**	4.38	0.02	4.43	0.02	0.0426	4.38	0.02	4.38	0.04	4.45	0.03	0.0691
**33_HEI2015C8_SEAPLANT_PR**	2.56	0.05	3.15	0.05	<.0001	2.56	0.05	2.93	0.09	3.23	0.06	<.0001
**34_HEI2015C9_FATTYACID**	4.52	0.08	4.94	0.09	0.0003	4.52	0.08	4.77	0.14	5.01	0.10	0.0004
**35_HEI2015C10_SODIUM**	4.47	0.07	4.83	0.07	<.0001	4.47	0.07	4.70	0.14	4.88	0.07	<.0001
**36_HEI2015C11_REFINEDGRA**	5.66	0.07	6.60	0.07	<.0001	5.66	0.07	6.21	0.13	6.75	0.09	<.0001
**37_HEI2015C12_SFAT**	4.84	0.08	5.41	0.09	<.0001	4.84	0.08	5.02	0.11	5.55	0.09	<.0001
**38_HEI2015C13_ADDSUG**	6.62	0.08	7.62	0.08	<.0001	6.62	0.08	7.12	0.12	7.81	0.08	<.0001

Supplemental Table S2. Comparison of nutritional scores (HEI and NRF) between consumers and non-consumers of healthy grains and between tertiles of consumption established with NRF9.3g. Raw scores and p values.

.

	Non-consumers and consumers of health grains (NRF9.3g based)					Tertiles of consumptions of healthy grains (NRF9.3g based)						
	NC		C			T1 (≤105g/d)		T2 (105-525g/d)		T3 (>525g/d)		
	Mean	SE	Mean	SE	P	Mean	SE	Mean	SE	Mean	SE	P
**0_DRXTKCAL**	1960.50	19.50	2061.57	10.37	<.0001	1954.00	19.18	1958.63	16.69	2174.47	15.40	<.0001
**10_NRF9**	406.25	5.37	474.14	3.78	<.0001	408.66	5.19	462.86	5.02	486.72	3.55	<.0001
**11_NR**	540.30	3.39	589.85	2.58	<.0001	541.63	3.24	581.52	3.45	599.55	2.62	<.0001
**12_ratio_p_prot**	98.86	0.11	99.32	0.05	<.0001	98.89	0.10	99.37	0.07	99.28	0.08	0.0003
**13_ratio_p_fibre**	51.99	0.67	57.96	0.52	<.0001	52.10	0.61	56.24	0.66	59.87	0.57	<.0001
**14_ratio_p_vitA**	52.57	0.81	62.76	0.61	<.0001	52.81	0.80	61.23	0.67	64.62	0.82	<.0001
**15_ratio_p_vitC**	59.56	0.89	64.54	0.70	<.0001	59.81	0.89	65.01	0.89	64.12	0.78	<.0001
**16_ratio_p_vitD**	19.04	0.42	23.60	0.32	<.0001	19.29	0.43	23.15	0.39	24.06	0.47	<.0001
**17_ratio_p_calcium**	64.58	0.64	70.05	0.41	<.0001	64.65	0.61	68.95	0.59	71.36	0.37	<.0001
**18_ratio_p_iron**	61.53	0.37	74.38	0.30	<.0001	61.70	0.36	70.26	0.40	78.99	0.47	<.0001
**19_ratio_p_potassium**	67.33	0.56	69.49	0.35	0.0003	67.50	0.53	70.18	0.54	68.77	0.39	0.0009
**20_ratio_p_magnesium**	64.85	0.59	67.75	0.38	<.0001	64.88	0.56	67.14	0.57	68.48	0.37	<.0001
**21_LIM**	134.05	2.59	115.71	1.69	<.0001	132.97	2.63	118.66	2.26	112.83	1.66	<.0001
**22_ratio_n_add_sugar**	48.66	2.40	40.48	1.36	0.0008	48.27	2.41	39.79	1.84	41.06	1.60	0.0033
**23_ratio_n_sfa**	39.66	0.96	34.97	0.77	<.0001	39.42	1.00	38.18	0.80	31.78	0.90	<.0001
**24_ratio_n_sodium**	45.73	1.13	40.27	0.54	<.0001	45.27	1.10	40.69	0.87	39.99	0.67	0.0001
**25_HEI2015_TOTAL_SCORE**	50.18	0.46	53.24	0.41	<.0001	50.38	0.45	52.93	0.49	53.52	0.43	<.0001
**26_HEI2015C1_TOTALVEG**	3.04	0.04	2.95	0.03	0.0660	3.04	0.04	3.07	0.04	2.82	0.04	<.0001
**27_HEI2015C2_GREEN_AND_B**	1.89	0.06	1.81	0.06	0.2363	1.89	0.06	1.84	0.07	1.77	0.07	0.1917
**28_HEI2015C3_TOTALFRUIT**	1.98	0.06	2.39	0.05	<.0001	2.00	0.06	2.36	0.07	2.41	0.04	<.0001
**29_HEI2015C4_WHOLEFRUIT**	2.13	0.07	2.67	0.05	<.0001	2.16	0.06	2.66	0.07	2.69	0.05	<.0001
**30_HEI2015C5_WHOLEGRAIN**	1.55	0.07	3.17	0.06	<.0001	1.58	0.06	2.61	0.06	3.78	0.08	<.0001
**31_HEI2015C6_TOTALDAIRY**	4.87	0.07	5.29	0.05	<.0001	4.88	0.07	5.27	0.06	5.34	0.07	<.0001
**32_HEI2015C7_TOTPROT**	4.49	0.03	4.37	0.02	<.0001	4.49	0.03	4.45	0.03	4.27	0.03	<.0001

**33_HEI2015C8_SEAPLANT_PR**	2.72	0.06	2.88	0.05	0.0081	2.74	0.06	2.89	0.05	2.86	0.06	0.0565
**34_HEI2015C9_FATTYACID**	4.83	0.10	4.67	0.07	0.1143	4.86	0.10	4.69	0.09	4.61	0.08	0.1142
**35_HEI2015C10_SODIUM**	4.47	0.09	4.72	0.06	0.0079	4.49	0.09	4.70	0.10	4.73	0.07	0.0479
**36_HEI2015C11_REFINEDGRA**	6.46	0.08	5.94	0.06	<.0001	6.47	0.08	6.27	0.09	5.57	0.08	<.0001
**37_HEI2015C12_SFAT**	4.80	0.09	5.24	0.08	<.0001	4.83	0.10	4.89	0.09	5.58	0.09	<.0001
**38_HEI2015C13_ADDSUG**	6.94	0.10	7.15	0.07	0.0269	6.95	0.10	7.23	0.09	7.09	0.08	0.0353

Supplemental Table S3. Comparison of nutritional scores (HEI and NRF) between consumers and non-consumers of healthy grains and between tertiles of consumption established with CFQS3. ADJUSTED MEANS and ADJUSTED PVAL. Adjusted variables : energy intakes (except for energy intake), age, gender, ethnicity and IPR.

	**Non-consumers and consumers of health grains (CFQ3 based)**					**Tertiles of consumptions of healthy grains (CFQS3 based)**						
	**NC**		**C**			**T1**		**T2**		**T3**		
	**Mean**	**SE**	**Mean**	**SE**	**P**	**Mean**	**SE**	**Mean**	**SE**	**Mean**	**SE**	**P**
**Amount of healthy grains (CFQS3), g/d**	0.00	0.00	76.71	2.22	<.0001	0.00	0.00	13.29	0.19	100.35	2.90	<.0001
**0_DRXTKCAL**	1880.99	11.57	1978.97	12.88	<.0001	1882.09	11.47	1861.12	24.01	2024.68	15.54	<.0001
**10_NRF9**	413.26	4.55	509.33	3.69	<.0001	413.50	4.52	464.64	5.61	526.65	4.16	<.0001
**11_NR**	543.79	3.03	608.96	2.52	<.0001	543.97	3.03	577.24	4.35	621.25	3.00	<.0001
**12_ratio_p_prot**	98.84	0.08	99.43	0.09	<.0001	98.84	0.08	99.38	0.13	99.45	0.10	<.0001
**13_ratio_p_fibre**	49.41	0.54	64.70	0.57	<.0001	49.46	0.53	56.16	0.69	68.01	0.66	<.0001
**14_ratio_p_vitA**	54.26	0.65	61.96	0.53	<.0001	54.27	0.65	60.05	0.94	62.71	0.67	<.0001
**15_ratio_p_vitC**	62.14	0.80	70.08	0.71	<.0001	62.16	0.80	67.05	1.40	71.26	0.70	<.0001
**16_ratio_p_vitD**	21.94	0.36	26.15	0.43	<.0001	21.95	0.36	24.70	0.60	26.71	0.53	<.0001
**17_ratio_p_calcium**	66.56	0.58	71.43	0.44	<.0001	66.57	0.58	68.95	0.77	72.40	0.52	<.0001
**18_ratio_p_iron**	67.98	0.38	75.44	0.42	<.0001	68.00	0.38	70.75	0.46	77.26	0.50	<.0001
**19_ratio_p_potassium**	63.68	0.39	69.84	0.44	<.0001	63.70	0.39	66.55	0.83	71.11	0.48	<.0001
**20_ratio_p_magnesium**	58.99	0.44	69.91	0.40	<.0001	59.02	0.44	63.65	0.65	72.34	0.47	<.0001
**21_LIM**	130.54	2.00	99.62	1.72	<.0001	130.47	1.98	112.60	2.35	94.60	1.79	<.0001
**22_ratio_n_add_sugar**	51.37	1.59	31.08	1.30	<.0001	51.32	1.58	40.18	2.27	27.55	1.33	<.0001
**23_ratio_n_sfa**	37.80	0.77	30.60	0.77	<.0001	37.79	0.77	33.56	1.04	29.45	0.81	<.0001
**24_ratio_n_sodium**	41.37	0.88	37.95	0.58	<.0001	41.36	0.88	38.85	1.28	37.60	0.69	<.0001
**25_HEI2015_TOTAL_SCORE**	47.15	0.37	56.77	0.34	<.0001	47.17	0.37	52.12	0.49	58.57	0.40	<.0001
**26_HEI2015C1_TOTALVEG**	2.61	0.03	2.86	0.04	<.0001	2.61	0.03	2.75	0.06	2.90	0.04	<.0001
**27_HEI2015C2_GREEN_AND_B**	1.56	0.06	1.99	0.05	<.0001	1.56	0.06	1.83	0.08	2.05	0.07	<.0001
**28_HEI2015C3_TOTALFRUIT**	2.23	0.05	2.86	0.06	<.0001	2.23	0.05	2.61	0.08	2.95	0.06	<.0001
**29_HEI2015C4_WHOLEFRUIT**	2.30	0.05	3.03	0.05	<.0001	2.30	0.05	2.78	0.08	3.13	0.05	<.0001
**30_HEI2015C5_WHOLEGRAIN**	1.06	0.05	4.66	0.06	<.0001	1.07	0.05	2.46	0.09	5.51	0.08	<.0001
**31_HEI2015C6_TOTALDAIRY**	5.20	0.06	5.49	0.07	0.0015	5.20	0.06	5.42	0.12	5.51	0.08	0.0062

**32_HEI2015C7_TOTPROT**	4.24	0.02	4.29	0.03	0.0927	4.24	0.02	4.28	0.04	4.29	0.03	0.2388
**33_HEI2015C8_SEAPLANT_PR**	2.40	0.05	2.87	0.06	<.0001	2.40	0.05	2.72	0.09	2.93	0.06	<.0001
**34_HEI2015C9_FATTYACID**	4.39	0.08	4.84	0.07	0.0001	4.39	0.08	4.71	0.12	4.89	0.08	0.0002
**35_HEI2015C10_SODIUM**	4.72	0.08	4.98	0.06	0.0006	4.72	0.08	4.88	0.13	5.03	0.07	0.0009
**36_HEI2015C11_REFINEDGRA**	4.96	0.06	5.83	0.09	<.0001	4.96	0.06	5.48	0.12	5.97	0.10	<.0001
**37_HEI2015C12_SFAT**	4.95	0.08	5.65	0.08	<.0001	4.96	0.08	5.26	0.10	5.81	0.09	<.0001
**38_HEI2015C13_ADDSUG**	6.53	0.07	7.42	0.06	<.0001	6.54	0.07	6.94	0.11	7.60	0.07	<.0001

Supplemental Table S4. Comparison of nutritional scores (HEI and NRF) between consumers and non-consumers of healthy grains and between tertiles of consumption established with NRF9.3g. ADJUSTED MEANS and ADJUSTED PVAL. Adjusted variables : energy intakes (except for energy intake), age, gender, ethnicity and IPR.

	Non-consumers and consumers of health grains (NRF9.3g based)					Tertiles of consumptions of healthy grains (NRF9.3g based)						
	NC		C			T1		T2		T3		
	Mean	SE	Mean	SE	P	Mean	SE	Mean	SE	Mean	SE	P
**Amount of healthy grains (NRF9.3g), g/d**	0.00	0.00	77.21	1.29	<.0001	0.40	0.04	30.86	0.33	126.60	1.88	<.0001
**0_DRXTKCAL**	1833.79	14.76	1967.75	12.64	<.0001	1830.86	13.99	1881.29	20.21	2067.16	15.31	<.0001
**10_NRF9**	413.41	4.93	478.29	3.83	<.0001	415.11	4.74	462.29	4.39	496.86	4.24	<.0001
**11_NR**	541.33	3.12	589.00	2.59	<.0001	542.12	2.95	576.54	3.10	603.78	2.87	<.0001
**12_ratio_p_prot**	98.80	0.11	99.26	0.07	0.0002	98.83	0.10	99.30	0.06	99.22	0.11	0.0007
**13_ratio_p_fibre**	52.58	0.69	58.42	0.59	<.0001	52.60	0.67	56.01	0.62	61.17	0.67	<.0001
**14_ratio_p_vitA**	51.67	0.66	60.58	0.53	<.0001	51.84	0.64	58.62	0.61	62.97	0.74	<.0001
**15_ratio_p_vitC**	62.96	0.94	67.17	0.68	<.0001	63.10	0.91	67.07	0.83	67.42	0.84	<.0001
**16_ratio_p_vitD**	20.89	0.47	25.24	0.33	<.0001	21.10	0.47	24.59	0.38	25.98	0.50	<.0001
**17_ratio_p_calcium**	65.22	0.56	70.43	0.47	<.0001	65.22	0.51	68.90	0.56	72.30	0.52	<.0001
**18_ratio_p_iron**	62.35	0.38	75.44	0.32	<.0001	62.46	0.36	70.96	0.42	80.66	0.46	<.0001
**19_ratio_p_potassium**	65.09	0.51	67.25	0.33	0.0001	65.22	0.47	67.28	0.43	67.24	0.44	0.0006
**20_ratio_p_magnesium**	61.76	0.51	65.21	0.43	<.0001	61.75	0.48	63.81	0.53	66.83	0.48	<.0001
**21_LIM**	127.92	2.37	110.71	1.66	<.0001	127.01	2.37	114.24	2.01	106.92	1.95	<.0001
**22_ratio_n_add_sugar**	46.81	1.84	39.56	1.37	0.0018	46.57	1.86	39.76	1.62	39.12	1.82	0.0104
**23_ratio_n_sfa**	39.06	0.90	32.39	0.68	<.0001	38.87	0.88	36.15	0.70	28.39	0.88	<.0001
**24_ratio_n_sodium**	42.05	1.08	38.76	0.64	0.0017	41.58	1.03	38.33	0.87	39.42	0.82	0.0234
**25_HEI2015_TOTAL_SCORE**	49.77	0.40	52.55	0.37	<.0001	49.95	0.40	52.03	0.42	53.07	0.42	<.0001
**26_HEI2015C1_TOTALVEG**	2.78	0.04	2.71	0.03	0.1288	2.78	0.04	2.79	0.03	2.62	0.04	<0.0001
**27_HEI2015C2_GREEN_AND_B**	1.80	0.06	1.75	0.06	0.4548	1.80	0.06	1.74	0.07	1.74	0.07	0.6515
**28_HEI2015C3_TOTALFRUIT**	2.30	0.06	2.62	0.05	<.0001	2.31	0.06	2.57	0.07	2.68	0.05	<.0001
**29_HEI2015C4_WHOLEFRUIT**	2.36	0.07	2.77	0.05	<.0001	2.37	0.06	2.74	0.07	2.82	0.05	<.0001
**30_HEI2015C5_WHOLEGRAIN**	1.68	0.07	3.23	0.06	<.0001	1.70	0.06	2.65	0.07	3.88	0.09	<.0001
**31_HEI2015C6_TOTALDAIRY**	5.17	0.07	5.41	0.06	0.0043	5.17	0.06	5.42	0.05	5.43	0.08	0.0038
**32_HEI2015C7_TOTPROT**	4.32	0.03	4.24	0.02	0.0074	4.32	0.03	4.31	0.02	4.16	0.03	<.0001

**33_HEI2015C8_SEAPLANT_PR**	2.51	0.06	2.67	0.05	0.0071	2.54	0.07	2.66	0.05	2.66	0.06	0.0939
**34_HEI2015C9_FATTYACID**	4.62	0.09	4.59	0.06	0.7762	4.66	0.08	4.58	0.06	4.57	0.09	0.7191
**35_HEI2015C10_SODIUM**	4.80	0.08	4.87	0.07	0.4514	4.83	0.08	4.91	0.09	4.81	0.08	0.6452
**36_HEI2015C11_REFINEDGRA**	5.81	0.09	5.18	0.07	<.0001	5.83	0.08	5.55	0.07	4.76	0.09	<.0001
**37_HEI2015C12_SFAT**	4.82	0.09	5.48	0.07	<.0001	4.84	0.09	5.07	0.08	5.92	0.10	<.0001
**38_HEI2015C13_ADDSUG**	6.80	0.08	7.02	0.06	0.0170	6.80	0.08	7.04	0.08	7.02	0.08	0.0533
